# Characterization of Experimental Short-Fiber-Reinforced Dual-Cure Core Build-Up Resin Composites

**DOI:** 10.3390/polym13142281

**Published:** 2021-07-12

**Authors:** Eija Säilynoja, Sufyan Garoushi, Pekka K. Vallittu, Lippo Lassila

**Affiliations:** 1 Research Development and Production Department, Stick Tech Ltd.—Member of GC Group, 20520 Turku, Finland; 2 Department of Biomaterials Science and Turku Clinical Biomaterial Center—TCBC, Institute of Dentistry, University of Turku, 20500 Turku, Finland; sufgar@utu.fi (S.G.); pekka.vallittu@utu.fi (P.K.V.); lippo.lassila@utu.fi (L.L.); 3 City of Turku Welfare Division, Oral Health Care, 20101 Turku, Finland

**Keywords:** physical properties, fiber-reinforced resin, core-build-up material, fracture toughness

## Abstract

As a core build-up material, dual-cured (DC) resin-based composites are becoming popular. The aim of this research was to investigate specific physical and handling properties of new experimental short-fiber-reinforced DC resin composites (SFRCs) in comparison to different commercial, conventional DC materials (e.g., Gradia Core, Rebilda DC, LuxaCore Z, and Visalys^®^ CemCore). Degree of monomer conversion (DC%) was determined by FTIR-spectrometry using either self- or light-curing mode. The flexural strength, modulus, and fracture toughness were calculated through a three-point bending setup. Viscosity was analyzed at room (22 °C) and mouth (35 °C) temperatures with a rotating disk rheometer. The surface microstructure of each resin composite was examined with scanning electron microscopy (SEM). Data were statistically analyzed with analysis of variance ANOVA (*p* = 0.05). The curing mode showed significant (*p* < 0.05) effect on the DC% and flexural properties of tested DC resin composites and differences were material dependent. SFRC exhibited the highest fracture toughness (2.3 MPa m^1/2^) values and LuxaCore showed the lowest values (1 MPa m^1/2^) among the tested materials (*p* < 0.05). After light curing, Gradia Core and SFRCs showed the highest flexural properties (*p* < 0.05), while the other resin composites had comparable values. The novel DC short-fiber-reinforced core build-up resin composite demonstrated super fracture toughness compared to the tested DC conventional resin composites.

## 1. Introduction

As a consequence, of the improvement of adhesive dentistry, restoring strategies of severely damaged teeth have altered dramatically in recent years [1]. The conservation of tooth structure is becoming a key matter for clinicians as dentistry evolves to become less and less invasive. The strategy, from a biomimetic standpoint, is to replace missing tooth tissue with biomaterials that have similar physical properties, particularly in terms of fracture toughness, elastic modulus, and strength [2]. A popular biomimetic restorative technique recommends replacing enamel with glass or hybrid ceramic and dentine with conventional particulate filled composite or short-fiber-reinforced composite (SFRC) [3]. Many clinical reports support this restorative approach, which includes preparing a core build-up, with or without a root canal fiber post, and then placing indirect restorations [4,5]. The use of prefabricated fiber posts is recommended only in cases of severe loss of remaining coronal tooth structure to assist the retention of the final restoration and possibly to optimize the biomechanical behavior of the remaining tooth structure [5]. Different resin cements are utilized for post-luting procedures. The mechanical properties of these resin cements are less than those of dentine and posts, which might result in an area of high stress, especially when a thick layer of cement is present in a wide or flared canal, resulting in multiple crack formations and inadequate bonding [6]. Paradoxically, the weakest material of a restored tooth, i.e., the luting cement, is located at the area of a reconstructed tooth where the tensile stresses are highest during functioning of the tooth. Thus, with the present particulate filler containing luting cement, a restored tooth is not optimized from a biomechanical point of view.

In recent years, several manufacturers have developed resin composites with various flowability or viscosities that claimed (in combination with fiber posts) to restore structurally compromised endodontically treated teeth [7]. There are three main curing modes for these commercial core-build up and post cementation resins, namely self-curing, light-curing, and dual-curing. The latter was designed to compensate for the lack of photon accessibility in deep cavities, particularly in the pulp chamber and root canal while placing a post. They combine the quick and on-demand setup of light-curing materials with self-cure features. Most of these materials are made of methacrylate resin and have a higher filler content and mechanical strength compared to dual-cure resin cements [7]. It is important to know that adding extra particulate-fillers improves the mechanical properties of the post-luting agent but also increases the viscosity, which in turn affects the adhesive interface integrity and leads to a deterioration of the bond strength and the potential development of microleakage [8,9]. In addition, the high viscosity of the material may limit it from being injected into the root canal, resulting in gaps and voids at interfaces [9,10]. Conversely, high viscosity might offer benefits in the manipulation of the material during core-build up procedure.

Reinforcement of the resin composite with short glass fibers was one of the most effective approaches among the methods that have been studied to improve physical properties and fracture toughness [11,12]. Short fibers enhanced the ability of the material to resist crack initiation and propagation, by making it not only resilient, but also flexible and tough [13]. Consequently, the incorporation of micrometer scale glass fibers (microfibers) within dual-cure (DC) resin composite materials can enhance the fracture toughness of the materials without deteriorating the viscosity. Therefore, the goal of the current research was to investigate the effect of microfiber loading on specific physical and handling properties of an experimental DC core-build up resin composite to compare it with certain commonly used core-build up materials. To the best of our knowledge, little research exists in this field. Furthermore, the impact of self- and light-curing modes on the performance of the materials was also evaluated. The null hypotheses were that the material type, and curing protocol, will have no effect on the tested properties of DC core-build up resin composites.

## 2. Materials and Methods

The commercial and experimental DC resin composites with composition are shown in Table 1.

### 2.1. Flexural Strength and Modulus of Elasticity

Three-point bending test specimens (2 × 2 × 25 mm^3^) were prepared from each tested resin composite. Bar-shaped specimens were prepared in a half-split stainless steel mold between transparent Mylar sheets. Half of the specimens were polymerized using self-curing protocol and the other half polymerized using light-curing protocol. Light polymerization of the resin composite was done using a hand light-curing unit (D-Light^®^ Pro, GC Europe, Leuven, Belgium) for 20 s in five separate overlapping portions from both sides of the metal mold. Light-tip of the curing device was 1 mm from the material. The wavelength of the light was between 400 and 480 nm and light intensity was 1200 mW/cm^2^ (Marc Resin Calibrator, Bluelight Analytics Inc., Halifax, NS, Canada). The specimens from each group (*n* = 8) were stored dry at 37 °C for 48 h before testing. A three-point bending test was conducted according to the ISO 4049 [14] (test span: 20 mm, crosshead speed: 1 mm/min, indenter: 2 mm diameter). All specimens were loaded in a material testing machine (model LRX, Lloyd Instrument Ltd., Fareham, UK) and the load-deflection curves were recorded with PC-computer software (Nexygen 4.0, Lloyd Instruments Ltd., Fareham, UK).

Flexural strength (ơ_f_) and flexural modulus (E_f_) were calculated from the following formula:

ơ_f_ = 3F_m_I/(2bh^2^) (1)

E_f_ = SI^3^/(4bh^3^) (2)

where F_m_ is the applied load (N) at the highest point of the load-deflection curve, I is the span length (20 mm), b is the width of the test specimens, and h is the thickness of the test specimens.

### 2.2. Fracture Toughness

Single-edge-notched-beam specimens (2.5 × 5 × 25 mm^3^), according to adapted ISO 20795-2 standard method (ASTM 2005), were prepared to determine the fracture toughness [15]. A custom-made stainless steel split mold was used, which enabled the removal of the specimen without force. An accurately designed slot was fabricated centrally in the mold, extending until its mid-height, which enabled central location of the notch and optimization of the crack length (x) to be half of the specimen’s height. The resin composite was inserted into the mold placed over a Mylar-strip-covered glass slide in one increment. Before polymerization, a sharp and centrally located crack was produced by inserting a straight edged steel blade into the prefabricated slot. Half of the specimens were polymerized using the self-curing protocol and the other half were polymerized using the light-curing protocol. Light polymerization of the resin composite was carried out for 20 s (D-Light^®^ Pro, GC Europe, Leuven, Belgium) in five separate overlapping portions. The upper side of the mold was covered with a Mylar strip and a glass slide from both sides of the blade, before being exposed to the polymerization light. Upon removal from the mold, each specimen was also polymerized on the opposite side. The specimens from each group (*n* = 6) were stored dry at 37 °C for 48 h before testing. The specimens were tested in three-point bending mode, in a universal material testing machine at a crosshead speed of 1.0 mm/min. The fracture toughness was calculated using the equation: Kmax = f(x) [P L/(B W^3/2^)]$\sqrt{10-3}$, where: f(x) = 3/2x^1/2^ [1.99 − x (1 − x) (2.15 − 3.93x + 2.7x^2^)]/2(1 + 2x) (1 − x)^3/2^ and 0 < x < 1 with x = a/W. Here, P is the maximum load in Newton (N), L is the span length (20 mm), B is the specimen thickness (mm), W is the specimen width (depth) in mm, x is a geometrical function dependent on a/W and, a is the crack length in mm.

### 2.3. Degree of Conversion

The degree of monomer conversion (DC%) of materials using self- and light-curing protocols was monitored by Fourier transform infrared spectroscopy (FTIR) (Spectrum One, Perkin-Elmer, Beaconsfield, UK) with an attenuated total reflectance (ATR) accessory. Resin composites were analyzed in a mold that was 2 mm thick and 5 mm in diameter. First, the spectrum of the unpolymerized sample was placed in the mold and measured. Then, FTIR spectra of self-curing groups were collected every minute up to 20 min after mixing. For light-curing groups, the sample was irradiated through an upper glass slide for 20 s with a visible light-curing unit (D-Light^®^ Pro, GC Europe, Leuven, Belgium). The sample was scanned for its FTIR spectrum after being irradiated. The DC% was calculated from the aliphatic C = C peak at 1638 cm^−1^ and normalized against the reference peak according to the following formula:

(3) $$\mathrm{DC}\%=\left[1-\frac{C_{aliphatic}/C_{reference}}{U_{aliphatic}/U_{reference}}\right]\times 100$$

where is the *C_aliphatic_* is the absorption peak at 1638 cm^−1^ of the cured specimen, *C_reference_* the reference peak of the cured specimen, *U_aliphatic_* is the absorption peak at 1638 cm^–1^ of the uncured specimen and *U_reference_* is the reference peak of the uncured specimen.

The fraction of remaining double bonds for each spectrum was determined by standard baseline techniques using the comparison of maximum heights of aliphatic and reference peaks for calculations. For each resin composite, five trials were performed per group.

### 2.4. Viscosity

Viscosity measurements were performed using a rotational rheometer (HAAKE RheoStress 300; Thermo Electron, Karlsruhe, Germany) applying shear stresses in dynamic oscillation mode with a parallel plate configuration (20 mm diameter). After DC resin composite was mixed and placed by automix tip on the lower plate of the rheometer, the upper plate was moved downward to adjust the gap to a thickness of 0.5 mm. Excess resin composite present around the circumference of the plate was removed prior to the measurements. All measurements were performed at 22 °C and 35 °C for 300 s and repeated three times. Viscosity of each DC resin composite after 15 s from mixing was calculated from the rheological measurement according to Equation:

*η* = *σ*/*γ̇* (4)

Here, *η* is apparent viscosity (Pa·s), *σ* is the shear stress (Pa), and *γ̇* is the shear rate (s^−1^) with a value of 1.

### 2.5. Microstructure Analysis

SEM and energy-dispersive spectroscopy (EDS) (GeminiSEM 450, Carl Zeiss, Oberkochen, Germany) provided the characterization of the microstructure of investigated DC resin composites. Polished specimen from each material was stored in desiccator for one day. Then, they were coated with a gold layer using a sputter coater in vacuum evaporator (BAL-TEC SCD 050 Sputter Coater, Balzers, Liechtenstein) before the SEM/EDS examination. SEM observations were carried out at an operating voltage of 20 kV and working distance of 10 mm.

### 2.6. Statistical Analysis

The resulted data were analyzed statistically using a two-way ANOVA test, followed by a one-way ANOVA and Tukey HSD test (*α* = 0.05) to illustrate the differences between the groups by SPSS version 23 (SPSS, IBM Corp., Armonk, NY, USA).

## 3. Results

**Figure 1:**
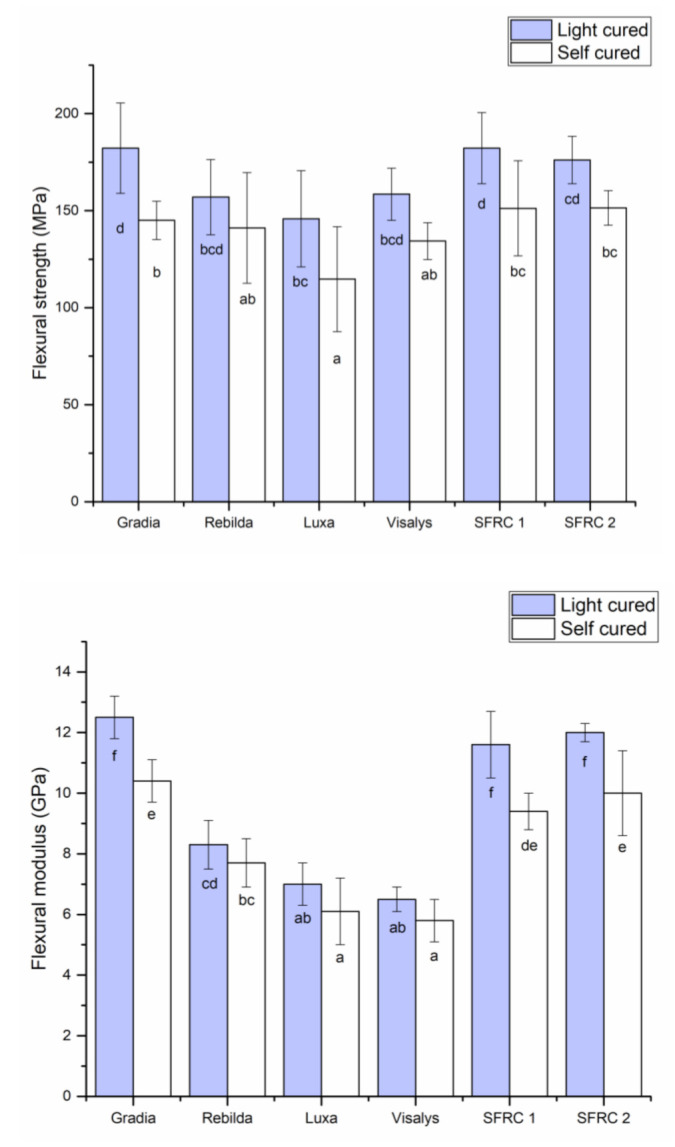
Bar graphs showing mean flexural strength (MPa) and flexural modulus (GPa) with standard deviations (SD) of tested DC resin composites. Groups denoted with the same letters are not statistically different (*p* < 0.05).

The results of flexural strength and flexural modulus of investigated DC resin composites are shown in Figure 1. The two-way ANOVA test revealed that material type and curing protocol significantly affected the tested flexural properties (*p* > 0.05). In general, the light-cure protocol always resulted in a significant improvement in flexural properties, regardless of the type of material. Gradia Core and SFRCs after light curing showed the highest flexural properties (*p* < 0.05), while the other resin composites had comparable values. No statistically significant differences were found in the flexural properties between SFRC1 and SFRC2 (Figure 1).

**Figure 2:**
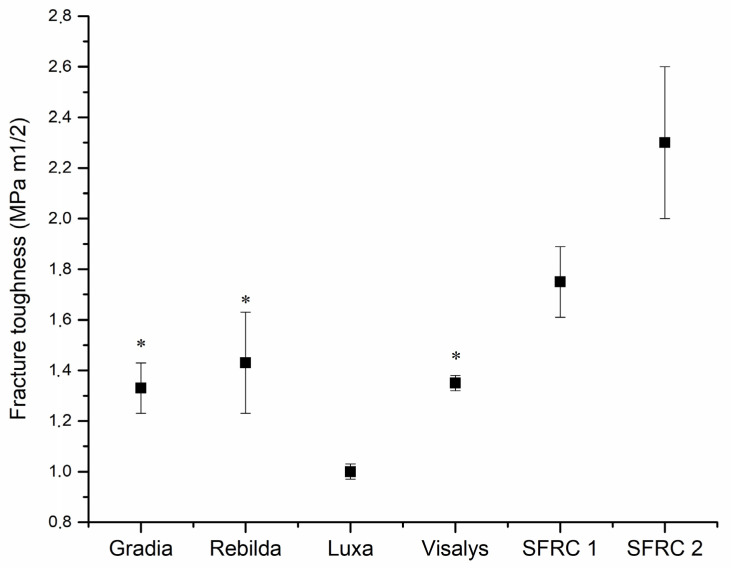
Graph showing mean fracture toughness (KIC) with standard deviations (SD) of investigated DC resin composites. The asterisk* above the boxes indicates statistically similar groups (*p* > 0.05).

Experimental SFRC2 exhibited the highest (*p* < 0.05) fracture toughness (2.3 MPa m^1/2^) values and LuxaCore showed the lowest values (1 MPa m^1/2^) among the tested materials (Figure 2).

**Figure 3:**
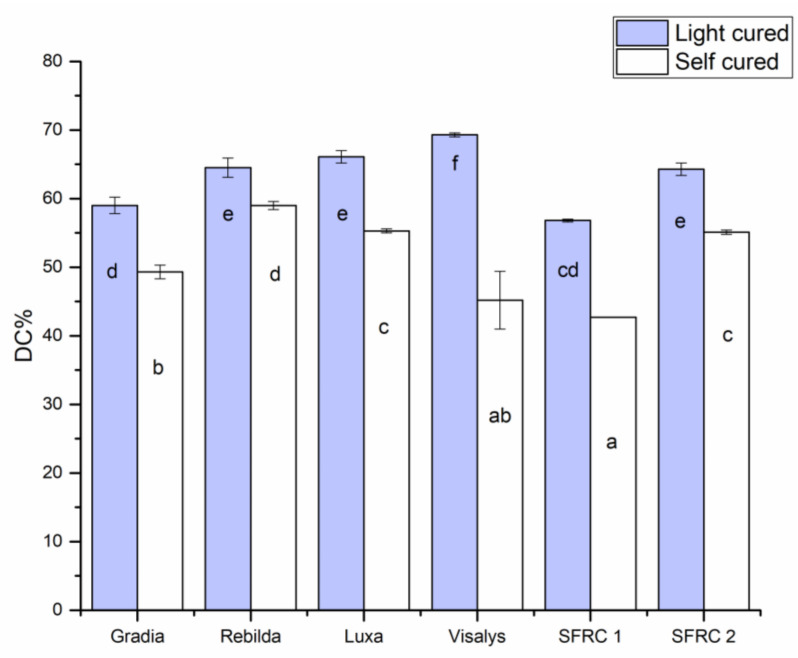
Bar graph illustrating the means of degree of conversion percentage (DC%) calculated at the bottom surface of tested DC resin composites. Groups denoted with the same letters are not statistically different (*p* < 0.05).

Degree of conversion (DC%) of resin composites significantly increased (*p* < 0.05) with light-curing protocol (Figure 3). DC% values of tested materials were a range between 42 and 69. After light-curing protocol, Visalys^®^ CemCore (Kettenbach GmbH & Co. KG, Eschenburg, Germany) showed the highest DC% (69) while SFRC1 showed the lowest values (59).

**Figure 4:**
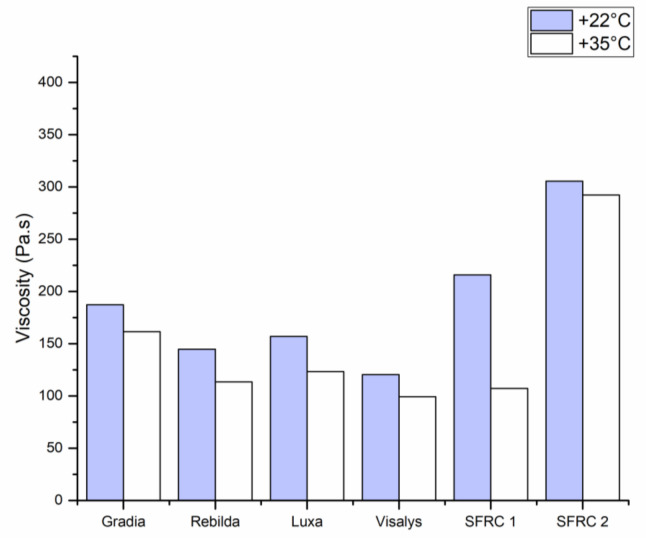
Bar graph illustrating the means of viscosity (Pa·s) of tested DC resin composites using rotational rheometer at 15 s after mixing with different temperatures.

A marked increase in viscosity was found for SFRC2 in both measuring temperatures (Figure 4). The higher temperature resulted in a reduction in viscosity for all tested materials.

Major elemental composition of each investigated resin composite determined with EDS is presented in Table 2.

**Figure 5:**
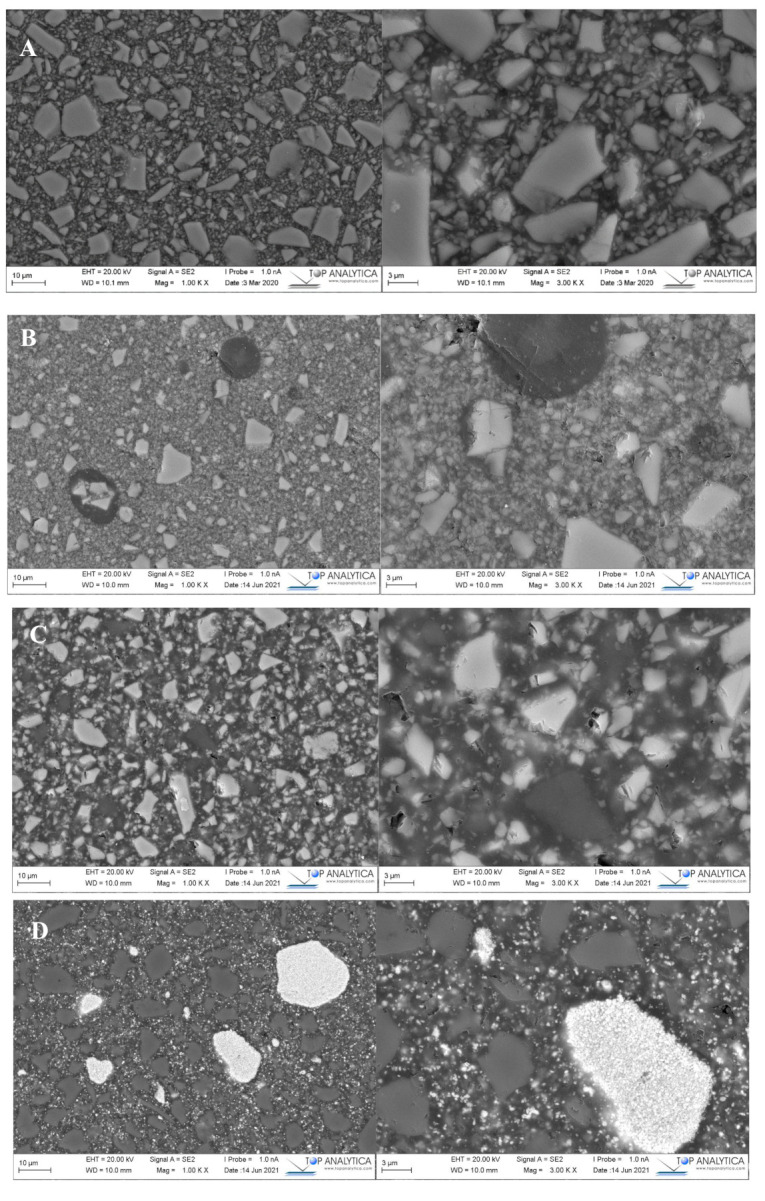

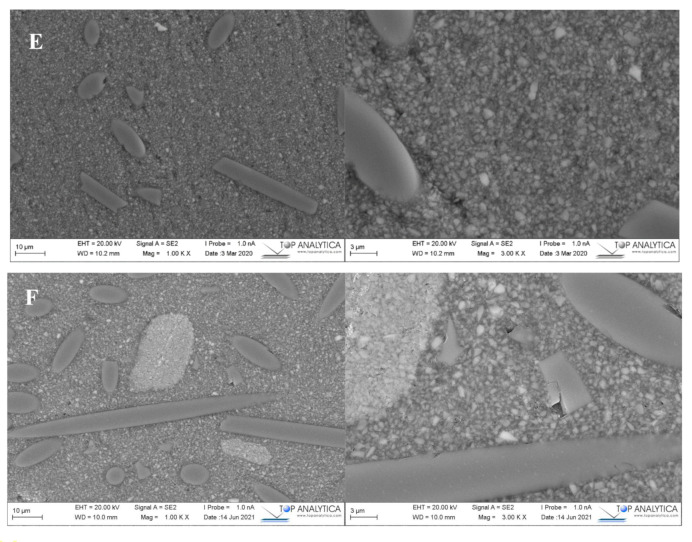
SEM photomicrographs (magnification: 1000 and 3000×) of polished surface of investigated materials (scale bar= 10 and 3 µm). (**A**) Gradia; (**B**) Rebilda; (**C**) Luxa; (**D**) Visalys^®^; (**E**) SFRC1; (**F**) SFRC2.

SEM analysis revealed homogenous and smooth surfaces where fillers are uniformly distributed in resin matrix (Figure 5). Specimens showed different particulate/fiber fillers size, shape, and quantity. This suggested an explanation for different toughening capability between tested materials. Chemical constituents of each investigated DC resin composite determined with EDS is presented in Table 2.

## 4. Discussion

In the current study, flexural properties and DC% were influenced by material type and curing mode. Hence, the null hypotheses were rejected. The three-point bending test (ISO Standard 4049) is routinely used in dental composites research because specimen preparation and load application are both straightforward and simple. Among the investigated DC resin composites, Gradia Core and SFRCs showed the highest values of flexural strength and modulus, which appears to be the outcome of high filler/fiber loading in comparison with the other tested materials. This is in good agreement with previous research that found a positive relationship between inorganic filler loading and flexural properties of resin composites [8,16]. However, the differences in flexural properties among the investigated resin composites might be attributed to variables other than filler loading (Table 2 and Figure 5). There may be variations in filler type, size, resin matrix composition, filler silanization quality, or the existence of some crack-resistant structures such as fibers and nano-clustering of fillers [17]. According to analysis of the EDS results, the investigated materials within the same class only have minor differences present on some oxides (Table 2), suggesting that the difference in the performance could be mainly dependent on the morphology and distribution of the reinforcing fillers. However, only major particles are analyzed, and no final conclusions can be made based on them.

The tendency to favor a light-curing mode, as seen in this study, supports prior findings [7,18]. It can be noted that another type of dual-cure resin composite, i.e., luting resin composite, has previously shown a similar tendency [19,20]. Therefore, light curing is predicted to improve the strength of dual-cure resin composites where they are most needed, namely on the coronal side. Dentists must consider this improvement when constructing their composite core after material placement. However, despite the relative reduction in flexural strengths seen in the self-curing mode for the investigated resin composites, most of materials should work well in challenging regions, such as a root canal.

One of the most common causes of resin composite failure in clinical practice is fracture of the material or adjacent tooth structure [21]. Under occlusal stresses, micro-cracks propagate and spread through the various layers of the composite core restoration. Fracture toughness is a mechanical characteristic that defines the resistance of brittle materials to catastrophic propagation of cracks under an applied stress, therefore describing damage tolerance and serving as a measure of fatigue resistance [3]. It is clearly shown in literature that it was the brittleness of the conventional particle-reinforced materials that generated the bulk of fractures that propagated easily through the whole thickness of the restoration and reached adjacent teeth, allowing further crack propagation [22]. Thus, the basic characteristics of the presently used materials do not significantly prohibit fatigue crack propagation. It is of special importance to note that damage caused by tensile stress while restoring a tooth is high especially at the area of the outer surface of the tooth; thus, the tougher material should be located here rather than in the middle of the root canal where the neutral axis of stress is located. This approach, also called the individual fiber post concept, has been used successfully with continuous long glass fibers [23,24]. On the other hand, fiber-reinforced core composites showed the ability to re-direct and stop crack propagation within the materials [25]. The presence of such energy-absorbing and stress-distributing fibers allows crack propagation to be deflected away from the bulk of the material and toward the peripheries [22,26,27].

Our data showed that dual-cure SFRCs had the highest fracture toughness values among the tested materials (Figure 2). Remarkably and because of fiber/filler loading differences, SFRC2 (2.3 MPa m^1/2^) had higher fracture toughness than SFRC1 (1.7 MPa m^1/2^). The effectiveness of fiber reinforcement is determined by a variety of factors including: the resins used; the weight, orientation; location of the fibers; the aspect ratio; the adhesion of fiber to the polymer matrix; and the impregnation of fiber into the resin [28].

In this research, we employed ATR-FTIR to track the DC% of resin composites with time. The key benefit of ATR-FTIR is that it can directly determine the DC% of resin composite after mixing. We observed the same trend as was apparent with flexural properties, where curing mode significantly affected the DC% of tested resin composites (Figure 3). It is crucial to note that the DC% measurements in this study were taken at room temperature rather than at 35 °C. Temperature has been shown to have a substantial impact on the DC% of resin composites, especially when resins completely self-cure [19,29]. As shown in Table 1, some of the used materials were BisGMA-based, and some were UDMA-based dual-cure resin composites. Since UDMA-based resin composites do not include an aromatic ring, to determine the DC% for those materials, another reference peak has to be selected. We utilized the secondary amide peak at 1530 cm^−1^ to measure DC% of the UDMA-based resin composites [30]. Because of this variation and measuring environment (room temperature), it may be preferable not to directly compare the DC% findings between the tested resin composites.

The rheological characteristics of investigated dual-cure resin composites were evaluated using a viscosity test. Data were reported at 15 s after mixing and with different temperatures, representing room and mouth conditions. Some of the used resin composites demonstrated a rapid rise in viscosity after mixing, which might be attributed to the starting of the resin polymer propagation process [31,32]. The viscosity of the resin composite increased after mixing because the polymerization reaction had started: when the polymer chains become longer and cross-linked to each other, viscosity becomes higher. A marked difference in viscosity was found among the investigated resin composites (Figure 4). The high viscosity of SFRC2 would be an influence of higher filler/fiber loading compared to other materials. The quantity of total fillers in SFRC1 accounts for 70% of its weight, whereas that in SFRC2 accounts for 75% of its weight. However, the high viscosity still might offer benefits in manipulation of the material during the core-build up procedure.

Temperature also influences viscosity. Directly after mixing, when materials are in a fluid paste, temperature’s influence can lead to more flexible molecular networks and, consequently, decreased viscosity [32]. This was clearly seen with SFRC1 when viscosity reduced by half after the temperature was raised to 35 °C. However, shortly after that, it was clear that the effect of temperature on the polymerization reaction acceleration overcame the effect of temperature on viscosity.

Further research is required to investigate the polymerization shrinkage properties (volumetric shrinkage and shrinkage stress) of these new dual-cure SFRCs, their adaptation and also their bonding efficiency, to dentine and fiber posts.

## 5. Conclusions

The experimental dual-cure short-fiber-reinforced flowable resin composites demonstrated enhanced fracture toughness compared with the conventional dual-cure resin composites. This might indicate the improved performance of SFRC as a core build-up and post-luting material in high stress-bearing applications. The self-curing protocol resulted in inferior results for some important material properties, regardless of material type.

## Figures and Tables

**Table 1:** Materials used.

Material	Manufacturer (Lot. No.)	Matrix Composition ^a^ (wt%)	Inorganic Filler Content ^a^ (wt%)
Gradia Core	GC Corp, Tokyo, Japan (1809041)	UDMA and other dimethacrylate (20–30)	alumino-silicate glass and silicon dioxide (70–75)
Rebilda DC	VOCO, Guxhaven, Germany (2003648)	Bis-GMA, DDDMA, UDMA (30)	Silanized silica (70)
LuxaCore Z	DMG, Hamburg, Germany (217782)	Bis-GMA and other dimethacrylate (15–45)	Silanized silica (55–75)
Visalys^®^ CemCore	Kettenbach GmbH & Co. KG, Eschenburg, Germany (20081)	UDMA and other dimethacrylate (20–50)	Ytterbium flourid and silica polymorph (50–70)
Short-fiber flowable composite (SFRC1)	GC Corp, Tokyo, Japan (Experimental)	UDMA and other dimethacrylate (30)	Short glass fiber (200–300 µm and Ø6 μm) + Barium glass (70)
Short-fiber flowable composite (SFRC2)	GC Corp, Tokyo, Japan (Experimental)	UDMA and other dimethacrylate (25)	Short glass fiber (200–300 µm and Ø6 μm) + Barium glass (75)

^a^ according to manufacturer’s information sheet. Bis-GMA, bisphenol-A-glycidyl dimethacrylate; TEGDMA, triethylene glycol dimethacrylate; UDMA, urethane dimethacrylate; DDDMA, 1,12-Dodecanediol dimethacrylate; Bis-EMA, ethoxylated bisphenol-A-dimethacrylate; wt%, weight percentage.

**Table 2:** Major elements of particles of investigated resin composites determined with EDS analysis.

Materials	Weight %
Gradia Core	C 37%, O 28%, Sr 13%, F 7.1%, Si 7.5%, Al 7%, Ba 0.2%, P 0.4%
Rebilda DC	C 28.8%, O 33.6%, Al 3.5%, Si 21.9%, Ba 12.2%
LuxaCore Z	C 30.5%, O 32.9%, Al 3.2%, Si 21.5%, Ba 11.9%
Visalys^®^ CemCore	C 20.7%, O 22.5%, F 9.2%, Si 17.3%, Ge 0.8%, Yb 29.5%
SFRC1	C 27.9%, O 36%, F 1.7%, Al 4.4%, Si 19.5%, Ba 8.8%, Ca 1.4%
SFRC2	C 17%, O 32,9%, F 1.4%, Al 4.4%, Si 24%, Ca 1.2%, Ba 19%

## References

[1] Zarow M., Ramírez-Sebastià A., Paolone G., Porta J.D.R., Mora J., Espona J., Durán-Sindreu F., Roig M. (2017). A new classification system for the restoration of root filled teeth. Int. Endod. J..

[2] Zafar M.S., Amin F., Fareed M.A., Ghabbani H., Riaz S., Khurshid Z., Kumar N. (2020). Biomimetic Aspects of Re-storative Dentistry Biomaterials. Biomim.

[3] Keulemans F., Garoushi S., Lassila L., Vallittu P., Özcan M. (2017). Fillings and core-built ups. A Clinical Guide to Principles of Fibre Reinforced Composites in Dentistry.

[4] Skupien J.A., Cenci M.S., Opdam N.J., Kreulen C., Huysmans M.-C., Pereira-Cenci T. (2016). Crown vs. composite for post-retained restorations: A randomized clinical trial. J. Dent..

[5] Scotti N., Coero Borga F.A., Alovisi M., Rota R., Pasqualini D., Berutti E. (2012). Is fracture resistance of endodonti-cally treated mandibular molars restored with indirect onlay composite restorations influenced by fibre post insertion?. J. Dent..

[6] Uctasli S., Boz Y., Sungur S., Vallittu P., Garoushi S., Lassila L. (2021). Influence of Post-Core and Crown Type on the Fracture Resistance of Incisors Submitted to Quasistatic Loading. Polymers.

[7] Spinhayer L., Bui A., Leprince J., Hardy C. (2020). Core build-up resin composites: An in-vitro comparative study. Biomater. Investig. Dent..

[8] Koytchev E., Yamaguchi S., Shin-No Y., Suzaki N., Okamoto M., Imazato S., Datcheva M., Hayashi M. (2019). Comprehensive micro-mechanical characterization of experimental direct core build-up resin composites with different amounts of filler contents. Dent. Mater. J..

[9] Walcher J.G., Leitune V.C.B., Collares F.M., Balbinot G.D.S., Samuel S.M.W. (2019). Physical and mechanical properties of dual functional cements—an in vitro study. Clin. Oral Investig..

[10] Aksornmuang J., Nakajima M., Tagami J. (2014). Effect of viscosity of dual-cure luting resin composite core materials on bond strength to fiber posts with various surface treatments. J. Dent. Sci..

[11] Garoushi S., Säilynoja E., Vallittu P.K., Lassila L. (2013). Physical properties and depth of cure of a new short fiber reinforced composite. Dent. Mater..

[12] Vallittu P.K. (2015). High-aspect ratio fillers: Fiber-reinforced composites and their anisotropic properties. Dent. Mater..

[13] Lassila L., Keulemans F., Säilynoja E., Vallittu P.K., Garoushi S. (2018). Mechanical properties and fracture behavior of flowable fiber reinforced composite restorations. Dent. Mater..

[14] ISO 4049 (2019). Dentistry—Polymer-Based Filling and Restorative Materials.

[15] ASTM E1820-20b (2005). Standard Test Method for Measurement of Fracture Toughness.

[16] Garoushi S., Lassila L.V.J., Vallittu P.K. (2011). Influence of nanometer scale particulate fillers on some properties of microfilled composite resin. J. Mater. Sci. Mater. Electron..

[17] Oja J., Lassila L., Vallittu P., Garoushi S. (2021). Effect of Accelerated Aging on Some Mechanical Properties and Wear of Different Commercial Dental Resin Composites. Materials.

[18] Karakis D., Yıldırım-Bicer A.Z., Dogan A., Koralay H., Cavdar S. (2017). Effect of self and dual-curing on degree of conversion and crosslink density of dual-cure core build-up materials. J. Prosthodont. Res..

[19] I Nokoshi M., Nozaki K., Takagaki T., Okazaki Y., Yoshihara K., Minakuchi S., Eerbeek B.V.A.M. (2021). Initial curing characteristics of composite cements under ceramic restorations. J. Prosthodont. Res..

[20] Sulaiman T.A., Abdulmajeed A.A., Donovan T.E., Ritter A.V., Lassila L.V., Vallittu P.K., Närhi T.O. (2015). Degree of conversion of dual-polymerizing cements light polymerized through monolithic zirconia of different thickness-es and types. J. Prosthet. Dent..

[21] Opdam N.J., Bronkhorst E.M., Roeters J.M., Loomans B.A. (2007). A retrospective clinical study on longevity of poste-rior composite and amalgam restorations. Dent. Mater..

[22] Lassila L., Säilynoja E., Prinssi R., Vallittu P.K., Garoushi S. (2020). Fracture behavior of Bi-structure fiber-reinforced composite restorations. J. Mech. Behav. Biomed. Mater..

[23] Garoushi S., Tanner J., Keulemans F., Le Bell-Rönnlöf A.-M., Lassila L., Vallittu P.K. (2020). Fiber Reinforcement of Endodontically Treated Teeth: What Options Do We Have? Literature Review. Eur. J. Prosthodont. Restorat. Dent.

[24] Bijelic J., Garoushi S., Vallittu P.K., Lassila L.V.J. (2011). Fracture load of tooth restored with fiber post and experi-mental short fiber composite. Open Dent J..

[25] Fráter M., Sáry T., Jókai B., Braunitzer G., Säilynoja E., Vallittu P.K., Lassila L., Garoushi S. (2021). Fatigue behavior of endodontically treated premolars restored with different fiber-reinforced designs. Dent. Mater..

[26] Tiu J., Belli R., Lohbauer U. (2020). R-curve behavior of a short-fiber reinforced resin composite after water storage. J. Mech. Behav. Biomed. Mater..

[27] Tiu J., Belli R., Lohbauer U. (2020). Rising R-curves in particulate/fiber-reinforced resin composite layered systems. J. Mech. Behav. Biomed. Mater..

[28] Vallittu P.K. (2018). An overview of development and status of fiber-reinforced composites as dental and medical bi-omaterials. Acta Biomater. Odontol. Scandinavica..

[29] Oliveira M., Cesar P.F., Giannini M., Rueggeberg F.A., Rodrigues J., Arrais C.A. (2012). Effect of temperature on the degree of conversion and working time of dual-cured resin cements exposed to different curing conditions. Oper. Dent..

[30] Guerra R.M., Duran I., Ortiz P. (2008). FTIR monomer conversion analysis of UDMA-based dental resins. J. Oral Rehabil..

[31] Zeller D.K., Fischer J., Rohr N. (2021). Viscous behavior of resin composite cements. Dent. Mater. J..

[32] Pegoraro T., Fulgêncio R., Butignon L., Manso A., Carvalho R., Pegoraro T. (2015). Effects of Temperature and Aging on Working/Setting Time of Dual-cured Resin Cements. Oper. Dent..

